# Stationary Cycling Exercise With Virtual Reality to Reduce Depressive Symptoms Among People With Mild to Moderate Depression: Randomized Controlled Trial

**DOI:** 10.2196/72021

**Published:** 2025-07-15

**Authors:** Na Zhang, Chenlu Hong, Yuejia Wang, Haiqin Chen, Yueli Zhu, Miao Da, Zhongxia Shen, Xudong Zhao, Jiali Xu, Jiaxiu Sheng, Yanan Luo, Meiying Xu

**Affiliations:** 1 Huzhou Third Municipal Hospital, No. 2088 Tiaoxiao East Road, Wuxing District, Huzhou City, Zhejiang Province, Huzhou, Zhejiang, 313000, China, 86 13757289010; 2 School of Medicine and Nursing, Huzhou University, Huzhou, China; 3 School of Medicine and Nursing, Peking University, Beijing, China

**Keywords:** virtual reality, cycling, exercise therapy, depressive symptoms, randomized controlled trial, adherence

## Abstract

**Background:**

Virtual reality (VR) stationary cycling provides a potential solution to enhance adherence and reduce depressive symptoms, particularly for people with depression. However, high-quality evidence is needed to support its implementation in clinical practice.

**Objective:**

This study evaluated the clinical effects of a 12-week VR-based stationary cycling program on depressive symptoms in sedentary adults with mild to moderate depression.

**Methods:**

This study is a 12-week, 3-arm randomized controlled double-blind trial. Participants (aged 18‐60 years) with depression were recruited from a clinic and randomly assigned (1:1:1) to the VR high-intensity stationary cycling, VR moderate-intensity stationary cycling, or non-VR moderate-intensity stationary cycling group to receive face-to-face intervention. The primary outcome was the change in depressive symptoms measured by the Hamilton Depression Rating Scale (HAMD-17) between baseline and 12 weeks. Response rate, remission rate, satisfaction, compliance, and adverse events were assessed post intervention. Outcomes were analyzed using an ITT approach.

**Results:**

Between January 2023 and June 2024, a total of 114 participants were enrolled and randomly assigned to 3 groups (38 per group), with 101 completing the trial (33 in non-VR moderate-intensity stationary cycling, 35 in VR moderate-intensity cycling, and 33 in VR high-intensity cycling). All 114 randomly assigned participants were included in the ITT analysis. After the 12-week intervention, all groups showed significant improvements in depressive symptoms, as reflected by the mean difference in HAMD-17 scores within groups from baseline to week 12: non-VR moderate intensity (mean difference within groups: 10.9, 95% CI 9.3-12.5; response rate: 58%, remission rate: 40%); VR moderate intensity (mean difference within groups: 14.2, 95% CI 12.7-15.8; response rate: 84%, remission rate: 74%); and VR high intensity (mean difference within groups: 14.4, 95% CI 13.0-15.8; response rate: 92%, remission rate: 74%). VR-based groups showed greater reductions than the non-VR group (mean difference between groups: 3.3, 95% CI 1.9-4.9, for VR moderate-intensity; mean difference between groups: 3.9, 95% CI 2.5-5.2, for VR high intensity). No significant difference in symptom improvement was found between the 2 VR groups, although the moderate-intensity group reported higher satisfaction.

**Conclusions:**

The VR stationary cycling program is an efficacious health intervention for alleviating depressive symptoms in participants with mild to moderate depression. These findings could facilitate incorporating VR health intervention into clinical practice, enhance the response of cycling-based interventions, and improve patient adherence, supporting its use as a complementary treatment for depression.

## Introduction

Depression is a prevalent mental health issue globally, significantly impairing psychosocial functioning and diminishing quality of life. It is estimated that approximately 5% of adults experience depression [1], with a lifetime prevalence of 6.8% in China [2]. Depression poses a substantial global disease burden, being a leading cause of annual suicides and the primary contributor to disability worldwide [3].

Exercise training and physical activity are recognized as potential treatments for depression. As a nonpharmacological option, they offer benefits such as low cost, minimal side effects, and ease of implementation. They have been included in treatment guidelines as complementary therapies for mild to moderate depression [4]. Recent research increasingly supports the effectiveness of exercise as a treatment for depression [5-11]. Studies have shown that physical activity and exercise can promote the growth of new nerve cells and trigger the release of proteins such as brain-derived neurotrophic factor, which enhances the survival of nerve cells and helps alleviate depressive symptoms [12]. However, significant uncertainty remains regarding the effectiveness of exercise for depression. For example, the optimal intensity of exercise (whether moderate or high) [13-16] and the best types of exercise for depression treatment [7] still need further investigation.

Furthermore, when conducting exercise interventions for individuals with depression, poor adherence to training is a common challenge [17]. Virtual reality (VR) exercise training, as a digital health approach, simulates real-world experiences through devices such as smart exercise bikes and treadmills [18-20], which provides multisensory stimulation, diverse environments, and motivational feedback, helping to improve adherence compared with non-VR training. Research shows that VR exercise is often more engaging, efficient, and controllable than conventional methods [21,22]. However, evidence of its response in alleviating depression is inconsistent and lacks high-quality research [23].

Stationary cycling exercise is one of the most common forms of aerobic exercise, characterized by high safety, ease of operation, and widespread popularity. However, due to its monotonous nature, it can lead to boredom and poor adherence among patients [24]. VR-based stationary cycling exercise may help address this challenge, but currently, there is a lack of evidence regarding its intervention effects. This study aims to investigate the effects of 12 weeks of VR-based stationary cycling exercise on depressive symptoms in adults aged 18-60 years with a sedentary lifestyle. We hypothesize that the VR cycling intervention will significantly reduce depression scores compared with a non-VR control group, with higher-intensity VR exercise leading to better outcomes than moderate-intensity VR exercise. We also expect that participants will have greater adherence to and satisfaction with the VR cycling intervention than those in the non-VR group.

## Methods

### Trial Design and Procedure

This study is a 12-week, 3-arm randomized controlled double-blind design aimed to compare the effects of high-intensity VR stationary cycling, moderate-intensity VR stationary cycling, and moderate-intensity non-VR stationary cycling in adults with depression. Baseline and trial completion assessments will be conducted using the Hamilton Depression Rating Scale (HAMD-17). This trial follows the CONSORT-EHEALTH (Consolidated Standards of Reporting Trials of Electronic and Mobile Health Applications and Online Telehealth) guidelines (Checklist 1).

### Participants

This study recruited patients diagnosed with depression at the outpatient clinic of Huzhou Third Municipal Hospital, Zhejiang Province, China, from January 2023 to June 2024. The inclusion criteria for participants were (1) meeting the diagnostic criteria for mild to moderate depressive disorder (either depressive episode or recurrent depressive disorder with a current mild or moderate episode) according to the *International Statistical Classification of Diseases and Related Health Problems 10th Revision* (*ICD-10*), and a HAMD-17 score between 8 and 24; (2) aged 18-60 years; (3) educated at least to primary school level; (4) no regular exercise in the month before admission; and (5) no auditory or visual impairments and able to complete assessment scales. The exclusion criteria were (1) individuals with other psychiatric disorders (eg, individuals with bipolar disorder in a depressive episode); (2) those at high risk for motion sickness with severe dizziness, fainting, or seizures; (3) individuals with serious physical illnesses that prevent exercise; (4) pregnant or breastfeeding women; and (5) those with a strong aversion to exercise therapy. By limiting the study population to outpatients instead of inpatients as initially registered, we aimed to enhance the comparability of treatment effects and improve the validity of our findings. This adjustment was necessary because inpatients receive various treatment modalities, such as psychotherapy and physical therapy, which introduce potential confounding factors that are difficult to control and could bias the final results. Additionally, during initial recruitment, a substantial proportion of eligible participants were aged 45‐60 years. To ensure sufficient sample size and improve generalizability, we extended the upper age limit from 44 to 60 years. This adjustment was made prior to data analysis and was consistently applied across outpatient clinics.

### Ethical Considerations

This study was reviewed and approved by the ethics committee of Huzhou Third Municipal Hospital (no.: 2023 Lun Shen No. 046) in accordance with the Declaration of Helsinki and the Belmont Report. Informed consent was obtained from all participants after explaining the study’s purpose, procedures, potential risks and benefits, and alternative treatment options. Participation in this study was entirely voluntary, and participants had the right to refuse or withdraw at any time without providing a reason. To protect participant privacy, all data were anonymized or deidentified. Additionally, participants had the potential to benefit from the intervention, as it may effectively alleviate depressive symptoms, and they also received a transportation subsidy of 500 RMB (approximately US $78, based on the exchange rate at the time of the study) upon study completion.

### Randomization and Binding

This study is a randomized controlled double-blind design. Participants were randomly assigned to 1 of the 3 groups in a 1:1:1 randomization ratio (block size 4) using a computer-generated random sequence. The groups are as follows: intervention group 1 (VR High-Intensity Stationary Cycling+ Medication treatment), intervention group 2 (VR Moderate-Intensity Stationary Cycling+ Medication treatment), and control group (non-VR Moderate-Intensity Stationary Cycling+ Medication treatment). Random grouping was conducted by researchers who did not participate in patient assessment or data analysis. All assessors were blinded throughout the study even after the intervention and served by clinical psychiatric specialists. Patients in each group were not informed of their specific group assignments.

### Intervention

All VR and non-VR stationary cycling exercises took place at the rehabilitation exercise training room of the Huzhou Third Municipal Hospital, with clinical nurses providing guidance and monitoring. The exercise intensity was measured using a wristband heart rate and blood oxygen monitor from the VR equipment. The maximum heart rate (HR_max_) was calculated based on the American College of Sports Medicine’s classification standards (HR_max_=220 − Age), with moderate intensity defined as 64%-76% HR_max_ and high intensity defined as 77%-95% HR_max_. The resistance of the bike was adjusted during cycling to ensure that the patient’s heart rate remained within the target range. The specifics are as follows.

#### Non-VR Moderate-Intensity Stationary Cycling

Patients cycled on a stationary bike without VR for moderate intensity 3 times a week for 30 minutes each session. The Borg Rating of Perceived Exertion Scale was used to assess perceived exertion, with moderate intensity defined as a self-reported level of exertion that feels moderate or somewhat challenging.

#### VR Moderate-Intensity Stationary Cycling

Patients first performed 5 minutes of low-intensity stretching as a warm-up, including stretches for the back, hips, and hamstrings. Then, they used semi-immersive VR equipment connected to the stationary bike for moderate-intensity cycling, where they could control the virtual environment through pedaling to enhance sensory stimulation. The seat was adjusted according to the patient’s height to ensure proper pedaling mechanics, and heart rate and respiratory sensors were connected. Patients were guided to focus on the screen during cycling, with no other activities allowed. The cycling frequency was 3 times a week for 30 minutes, maintaining heart rates between 64% and 76% HR_max_. If discomfort such as excessive fatigue, chest tightness, or palpitations occurred, cycling was to be stopped immediately, and clinical nurses would provide timely assistance. After cycling, patients performed a 5-minute cooldown, which included techniques to relieve soreness in the lower back and legs through pressure relaxation or tapping with fists or palms. Tapping movements should start lightly and gradually increase in intensity and speed.

#### VR High-Intensity Stationary Cycling

The warm-up and cooldown were the same as those in the moderate-intensity VR group, but the cycling portion consisted of 30 minutes of intermittent high-intensity cycling (7 minutes on, 3 minutes off, and repeated 3 times). During cycling, patients maintained heart rates between 77% and 95% HR_max_, with the intervention frequency being 3 times a week over a total of 12 weeks.

#### VR Equipment

The VR equipment included a main unit, electronic display, and sensors for measuring heart rate, breathing, and speed, all connected to the stationary bike (Brand: Sunshine Heart Fitness Mental Adjustment Exercise System, Model: YG-SXTS-BZ). Patients could engage in semi-immersive VR cycling simulations via the electronic display, with 3 scenarios available: mountain roads, flower avenues, and roads, allowing patients to choose based on their preference. Additionally, the VR equipment included a wristband heart rate and blood oxygen monitor that displayed real-time heart rate and blood oxygen data, which could be wirelessly transmitted to the electronic display for patients to monitor their physiological indicators in real-time.

#### Medical Treatment

Patients received pharmacological treatment for depression using escitalopram oxalate (brand name: Lexapro, product of Lundbeck), starting at an initial dose of 5 mg per day. The dosage was gradually increased to an effective therapeutic dose of 10‐20 mg per day, administered once daily, for a continuous treatment duration of 12 weeks. Regarding medical treatment before participating in the study, 20 participants were experiencing their first depressive episode and had not taken any antidepressant medication, whereas 94 participants had been taking at least 1 type of antidepressant, with treatment durations ranging from 1 month to 9 years (mean 46, SD 23 months). Overall, medication use prior to study enrollment varied based on whether participants were experiencing their first depressive episode or had recurrent episodes.

### Primary Outcome

The primary outcome is the score on the depression scale, measured by the HAMD-17, assessed through web-based questionnaires. This scale was developed by Hamilton in 1960 and consists of 17 items. It has good reliability and validity in assessing depression in adult patients, with a Cronbach α coefficient of 0.714. A higher HAMD-17 score indicates a more severe level of depression in patients.

### Secondary Outcomes

The secondary outcomes include the response rate of depression, the remission rate of depression, exercise adherence, and patient satisfaction. The response rate is calculated as the number of responders in each group divided by the total number of cases in that group, multiplied by 100% (with response defined as a reduction of ≥50% in HAMD-17 scores after 3 months of exercise intervention). The remission rate is calculated as the number of remitted cases in each group divided by the total number of cases in that group, multiplied by 100% (with remission defined as a HAMD-17 score of ≤7 after 3 months of exercise intervention). Exercise adherence is calculated as recorded training completion times/required target times × 100%. Patients with an adherence percentage of less than 60% are classified as having poor adherence or moderate adherence, and those with an adherence percentage of ≥60% are classified as having good adherence or complete adherence. Patient satisfaction is collected using a self-developed satisfaction survey, which includes satisfaction regarding exercise training, intervention programs, exercise equipment, and intervention effects, rated on a 5-point scale, where 1 point indicates very dissatisfied and 5 points indicates very satisfied, with a total score of 20 points.

### Other Measurements

The safety indicator is measured by the adverse event incidence rate, where the adverse event incidence rate %=number of adverse events during the intervention/total number of participants in that group × 100%. Adverse events include sprains caused by cycling and physiological discomforts such as dizziness and vomiting caused by virtual scenarios, assessed through self-reporting by patients and observational methods. Covariates include the participants’ sex, age, education level, occupation, marital status, duration of illness, and dosage of antidepressants.

### Sample Size Estimate

The sample size estimation is based on the effect sizes (ESs) for the primary outcome, HAMD-17, derived from a prior pilot study with an ES of 0.35. In this pilot study, 10 participants were assigned to each group, with a total of 30 participants. The results showed that after 12 weeks of intervention, the average HAMD-17 scores for the 3 groups were 9, 6, and 5 for intervention group 1, intervention group 2, and the control group, respectively, with a within-group error mean square of 23.58 and a calculated ES of 0.35. Assuming a type I error rate of 5% (α) and 80% statistical power, at least 80 participants need to be recruited. After considering the dropout rate of 30%, a total of 114 subjects were recruited, with 38 participants each entering intervention group 1, intervention group 2, and the control group. Among them, 9 patients automatically withdrew from the intervention after baseline assessment, and 4 patients withdrew from the study due to worsening depressive symptoms, leaving a total of 101 patients who met the study requirements. The sample size calculation was estimated using G*Power (version 3.1.9.7; Heinrich Heine University Düsseldorf).

### Statistical Analysis

Descriptive analysis was used to compare the baseline demographic characteristics of the 3 groups. Ordered variables were presented using means and SDs or medians and IQRs, while categorical variables were presented using numbers and percentages. The characteristics of participants were compared using the chi-square test for categorical variables. After assessing the normality of continuous variables using the Shapiro-Wilk test and visual inspection of Q-Q plots, HAMD-17 scores and age were found to be nonnormally distributed; therefore, the Kruskal-Wallis test was used to assess differences in baseline demographic characteristics among the 3 groups. Primary outcomes were analyzed with an analysis of covariance model, with baseline data of participants’ sex, age, education level, occupation, marital status, duration of illness, and dosage of antidepressants as covariates, while the secondary outcome analysis was compared using the Kruskal-Wallis test. All analyses were performed in the intention-to-treat (ITT) strategy using multiple imputation, pooling results from 5 linear regression imputations. All statistical tests were 2-sided, with a significance level set at *P*<.05. All analyses were conducted using SPSS software (version 22.0; IBM Corp).

## Results

### Characteristics of Participants

**Figure 1.**
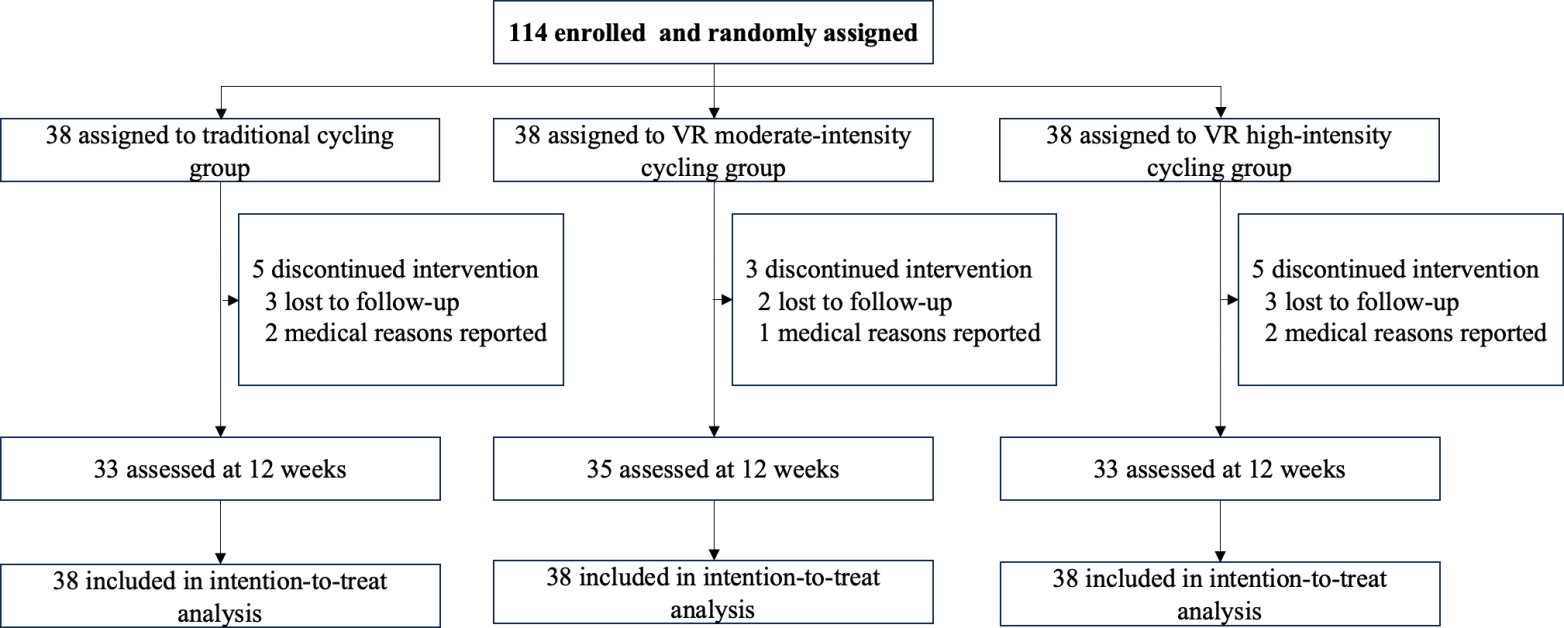
Trial profile. VR: virtual reality.

The flowchart of participants is shown in Figure 1. A total of 114 patients were enrolled from January 2023 to June 2024, and ultimately 101 patients completed the study. Among them, there were 38 cases in the traditional non-VR moderate-intensity cycling group (3 cases withdrew voluntarily and 2 cases of symptom relapse), 38 cases in the VR moderate-intensity cycling group (2 cases withdrew voluntarily and 1 case of symptom relapse), and 38 cases in the VR high-intensity cycling group (3 cases withdrew voluntarily and 2 cases of symptom relapse).

At baseline, there were no significant differences in the characteristics of sex, age, occupation, education level, marital status, and daily dosage of escitalopram oxalate among the non-VR Moderate-Intensity Stationary Cycling group, VR Moderate-Intensity Stationary Cycling group, and VR High-Intensity Stationary Cycling group. In addition, these 3 groups also had similar durations of depression diagnosis at baseline, with comparable HAMD-17 depression scores (non-VR Moderate-Intensity Stationary Cycling group: VR Moderate-Intensity Stationary Cycling group: VR High-Intensity Stationary Cycling group=20.33: 20.59: 20.11; *P*=.85). See Table 1.

**Table 1.** Baseline demographic data and outcome measure data.

Characteristics	Non-VR^a^ moderate-intensity stationary cycling (N=38)	VR moderate-intensity stationary cycling (N=38)	VR high-intensity stationary cycling (N=38)	Chi-square/Kruskal-Wallis H test^b^	*P* value
Age, years, mean (SD)	35.24 (13.00)	34.11 (13.25)	34.97 (13.00)	0.85 (2)	.65
Sex, n (%)				0.92 (2)	.63
Male	16 (42)	20 (53)	17 (45)		
Female	22 (58)	18 (47)	21 (55)		
Occupation, n (%)				1.79 (2)	.78
Workers and peasants	15 (40)	19 (50)	16 (42)		
Enterprises and governments	9 (24)	7 (18)	11 (29)		
Other	14 (37)	12 (26)	11 (29)		
Education, n (%)				0.71 (2)	.99
Primary school and below	8 (21)	11 (29)	10 (26)		
Junior school	10 (26)	9 (24)	10 (26)		
Senior school	13 (34)	12 (32)	12 (32)		
College and above	7 (18)	6 (16)	6 (16)		
Marital status, n (%)				0.42 (2)	.98
Married	27 (71)	25 (66)	25 (66)		
Unmarried	9 (24)	10 (26)	10 (26)		
Divorced or widowed	2 (5)	3 (8)	3 (8)		
Length of diagnosed depression, mean (SD)	3.50 (3.00)	3.63 (2.25)	4.36 (2.00)	3.69 (2)	.16
Escitalopram oxalate (mg/d), mean (SD)	15.66 (6.00)	15.52 (6.25)	15.13 (6.25)	0.46 (2)	.80
HAMD-17^b^, mean (SD)	20.33 (6.00)	20.59 (4.50)	20.11 (6.00)	0.27 (2)	.85

a VR: virtual reality.

b HAMD-17: Hamilton Depression Rating Scale.

### Intervention Effect

Compared with baseline, all groups—non-VR Moderate-Intensity Stationary Cycling group, VR Moderate-Intensity Stationary Cycling group, and VR High-Intensity Stationary Cycling group—showed a significant reduction in HAMD-17 scores after 12 weeks of intervention. In the non-VR Moderate-Intensity Stationary Cycling group, the HAMD-17 score decreased significantly by 10.87 (95% CI 9.28-12.46). The HAMD-17 score in the VR Moderate-Intensity Stationary Cycling group decreased significantly by 14.22 (95% CI 12.65-15.79), while the VR High-Intensity Stationary Cycling group saw a significant decrease of 14.43 (95% CI 13.01-15.84) (Table 2).

**Table 2.** Primary outcomes in the intention-to-treat population^a^.

Characteristics	N	Baseline	Week 12	Mean difference in change between baseline and week 12, mean (95% CI)	Mean difference in change between groups, mean (95% CI)	*P* value	Cohen *d*/η²^b^
HAMD-17^c^, mean (SD)							0.24 (η²)
Non-VR^d^ moderate-intensity stationary cycling	38	20.33 (6.00)	9.05 (7.00)	10.87 (9.28 to 12.46)	N/A^e^	N/A	N/A
VR moderate intensity cycling	38	20.59 (4.50)	6.16 (3.00)	14.22 (12.65 to 15.79)	3.27 (1.91 to 4.93)	<.001	0.65 (Cohen *d*)
VR high-intensity stationary cycling	38	20.11 (6.00)	6.34 (3.25)	14.43 (13.01 to 15.84)	3.87 (2.52 to 5.22)	<.001	0.69 (Cohen *d*)
VR moderate-intensity versus VR high-intensity	N/A	N/A	N/A	N/A	0.60 (−1.06 to 2.27)	.67	0.13 (Cohen *d*)

a Data are mean (SD).

b Cohen *d* values represent effect sizes for comparisons between each VR group and the non-VR group, or between the VR moderate- and high-intensity groups. η² represents the effect size from ANOVA for the comparison among all 3 groups.

c HAMD-17: Hamilton Depression Rating Scale.

d VR: virtual reality.

e Not applicable.

Compared with the non-VR Moderate-Intensity Stationary Cycling group, the VR Moderate-Intensity Stationary Cycling group and the VR High-Intensity Stationary Cycling group showed greater reductions in HAMD-17 scores by 3.27 (95% CI 1.91-4.93) and 3.87 (95% CI 2.52-5.22), respectively, with an ES of 0.65 (*P*<.001) and 0.69 (*P*<.001), respectively. The VR Moderate-Intensity Stationary Cycling group and the VR High-Intensity Stationary Cycling group showed greater responsiveness than the non-VR Moderate-Intensity Stationary Cycling group in reducing depression scores. However, the intervention effects of the VR Moderate-Intensity Stationary Cycling group and the VR High-Intensity Stationary Cycling group were similar; compared with the VR High-Intensity Stationary Cycling group, the HAMD-17 score in the VR Moderate-Intensity Stationary Cycling group decreased by 0.60 (95% CI −1.06 to 2.27), but the difference was not significant. More details can be found in Table 2.

### Response Rate, Remission Rate, Satisfaction, Adherence, and Adverse Events

After the 12-week intervention, the VR moderate-intensity cycling group demonstrated a response rate of 84% and a remission rate of 74%. In comparison, the VR high-intensity cycling group achieved a response rate of 92% and a remission rate of 74%. Both VR groups outperformed the non-VR Moderate-Intensity Stationary Cycling group, which recorded a response rate of 58% and a remission rate of 39%, with the differences being statistically significant (Table 3). However, the differences in response and remission rates between the VR moderate-intensity and VR high-intensity cycling groups were not statistically significant. Further details can be found in Table 3.

**Table 3.** Response rate, remission rate, patient satisfaction, and exercise adherence among participants^a^.

	N	Week 12	Chi-square/Kruskal-Wallis H test	*P* value
Response rate, n (%)			14.24 (2)	.001
Non-VR Moderate-Intensity Stationary Cycling	38	22 (58)	N/A^b^	N/A
VR Moderate-Intensity Stationary Cycling	38	32 (84)	6.40^c^ (2)	<.05
VR High-Intensity Stationary Cycling	38	35 (92)	11.86^d^ (2)	.001
Remission rate, n (%)			12.62 (2)	<.05
Non-VR Moderate-Intensity Stationary Cycling	38	15 (39)	N/A	N/A
VR Moderate-Intensity Stationary Cycling	38	28 (74)	9.05^e^ (2)	<.05
VR High-Intensity Stationary Cycling	38	28 (74)	9.05^f^ (2)	<.05
Patient satisfaction, mean (SD)			41.03 (2)	<.001
Non-VR Moderate-Intensity Stationary Cycling	38	11.10 (4.00)	N/A	N/A
VR Moderate-Intensity Stationary Cycling	38	16.09 (4.00)	48.41^g^ (2)	<.001
VR High-Intensity Stationary Cycling	38	13.64 (5.00)	24.86^h^ (2)	<.05
Exercise adherence, n (%)			12.89 (2)	<.05
Non-VR Moderate-Intensity Stationary Cycling	38	18 (49)	N/A	N/A
VR Moderate-Intensity Stationary Cycling	38	30 (81)	11.19^i^ (2)	<.05
VR High-Intensity Stationary Cycling	38	27 (73)	6.17^j^ (2)	.10

a Data are presented as mean (SD) or n (%). Exercise adherence was calculated as recorded training completion times/required target times ×100%. Patients with an adherence percentage of ≥60% were classified as having complete adherence or good adherence.

b Not applicable.

c VR moderate-intensity versus non-VR moderate-intensity (response rate).

d VR high-intensity versus non-VR moderate-intensity (response rate).

e VR moderate-intensity versus non-VR moderate-intensity (remission rate).

f VR high-intensity versus non-VR moderate-intensity (remission rate).

g VR moderate-intensity versus non-VR moderate-intensity (satisfaction).

h VR high-intensity versus non-VR moderate-intensity (satisfaction).

i VR moderate-intensity versus non-VR moderate-intensity (adherence).

j VR high-intensity versus non-VR moderate-intensity (adherence). No statistically significant differences were found between the VR moderate-intensity and VR high-intensity cycling groups in response rate (*χ*2=0.10; *P*=.75), remission rate (*χ*2=0.88; *P*=.34), and adherence rate (*χ*2=2.55; *P*=.47), while patient satisfaction was significantly higher in the in VR moderate-intensity group than in the VR high-intensity group (*H*=23.55; *P*=.01).

Patient satisfaction in the VR moderate-intensity cycling group was significantly higher than that in both the VR high-intensity cycling group and the non-VR Moderate-Intensity Stationary Cycling group, with these differences being statistically significant. Moreover, the satisfaction levels of patients in the VR high-intensity cycling group were higher than those in the Non-VR Moderate-Intensity Stationary Cycling group. More information is available in Table 3.

Compliance levels among patients in the VR moderate-intensity cycling group exceeded those of the non-VR Moderate-Intensity Stationary Cycling group, resulting in a statistically significant difference (*χ*^2^=11.19; *P*=.01). However, the VR high-intensity cycling group did not show a statistically significant difference in compliance when compared with either the VR moderate-intensity group or the non-VR Moderate-Intensity Stationary Cycling group. Additional details are shown in Table 3.

None of the patients in any of the 3 groups reported experiencing any adverse events (including riding-related injuries or physiological discomfort, such as dizziness or nausea due to the virtual environment). Prior estimates from clinical experience and previous research suggested a 30% expected dropout rate, whereas the observed overall rate was lower at 11%, with group-specific variations: 13% in traditional cycling (3 voluntary withdrawals and 2 symptom relapse), 8% in VR moderate-intensity cycling (2 voluntary withdrawals and 1 symptom relapse), and 13% in VR high-intensity cycling (3 voluntary withdrawals and 2 symptom relapse). There were no obvious differences in characteristics between participants who completed the 12-week outcome assessment and participants who did not (Table S1 in Multimedia Appendix 1). The Last Observation Carried Forward approach to missing data offers a conservative estimate of the individual outcome in a study, and the sample size for the study allowed for the expected dropout rate.

## Discussion

To our knowledge, this study is among the few to evaluate the effects of VR-based stationary cycling on depression levels in individuals with mild to moderate depression in a low-income country. Our findings indicated that both VR-based and traditional stationary cycling significantly reduced the depression level indicators of the patients, emphasizing the therapeutic value of stationary cycling exercise. Furthermore, compared with the traditional stationary cycling, the VR-based intervention led to a significantly greater reduction in HAMD-17 scores after 12 weeks, suggesting that this multimodal VR approach enhanced the effectiveness of stationary cycling interventions.

Our findings indicated that VR-based cycling is more effective than traditional cycling in improving depression levels, which is consistent with previous studies. A systematic review previously found that VR-based exercise games had stronger effects on depression outcomes than other outcomes such as cognitive function and memory. One potential reason is that depression often diminishes motivation, necessitating supervision and encouragement to sustain physical activity [25], and VR enhances engagement by creating an immersive environment, diverting attention from physical discomfort, increasing enjoyment, and fostering positive emotions toward exercise [24,26,27]. These factors improve adherence, which may contribute to better therapeutic outcomes. Our results support this, as participants in the VR-based cycling group reported higher adherence and satisfaction than those in the traditional cycling group. Additionally, prior studies suggest that VR’s interactive nature may implicitly enhance cognitive skills such as attention and planning, further benefiting depression management [28]. For individuals with depression, VR-enhanced exercise integrates physical activity with immersive VR technology through motion-tracked adaptive environments [29,30], which may contribute to (1) regulate mood by simulating natural settings that provide a relaxing exercise space, potentially modulating stress-related biomarkers (eg, cortisol and serotonin), and (2) induce cognitive distraction by diverting attention from physical discomfort via interactive tasks, thereby reducing perceived exertion. Additionally, compared with other digital interventions, such as cognitive behavioral therapy, research has shown that the response rate of cognitive behavioral therapy is 82.35% which is lower than that in moderate-intensity or high-intensity VR cycling [31]. VR cycling offers a more immersive experience and greater interactivity, leading to better engagement and adherence, but the high cost and limited accessibility for at-home use remain significant challenges.

Contrary to our initial hypothesis, no significant difference was found between the VR high-intensity and moderate-intensity cycling groups in terms of depression score reductions, which is consistent with previous mixed results [32,33]. This lack of difference may be attributed to 2 reasons. First, previous research suggests that the antidepressant effects of exercise may be mediated through anti-inflammatory mechanisms [34]. While moderate-intensity exercise intervention reduces proinflammatory cytokine levels, high-intensity exercise increases the levels of inflammatory factors such as tumor necrosis factor-α and interleukin-6 [33,35]. Second, for individuals with depression, high-intensity exercise may induce greater physical stress, exacerbating stress levels, whereas moderate-intensity exercise fosters self-esteem and a sense of control [36-38]. Additionally, the relatively short duration of our intervention may not have been sufficient to detect significant differences between intensity levels. However, we found that patient satisfaction was higher in the VR moderate-intensity cycling group than that in the VR high-intensity group, potentially due to the greater physical and psychological stress associated with high-intensity training [39].

This study has several limitations. First, the practice effect may have influenced our results, although it is challenging to separate it from the intervention effect. Future studies could use alternative testing methods to minimize this bias. Second, while our clinician-administered VR cycling demonstrated strong feasibility, the single-region study design in China may limit generalizability to broader populations. Future studies could enhance the validation of our results by conducting multicenter trials and cost-effectiveness analysis. Third, although our active-control design (comparing VR cycling with traditional cycling) ensures that all participants received treatment, the absence of a nonexercise control group makes it challenging to determine the absolute treatment effect against standard care. Future research should include a nonexercise control group to better evaluate the intervention’s effects. Fourth, given the potential benefits of personalized VR experiences, future studies should investigate VR customization to improve adherence and optimize outcomes. Finally, the study did not assess long-term adherence, and the sustainability and continued effectiveness of VR cycling postintervention remain unclear. Future research will focus on follow-up to evaluate long-term effects.

In summary, our study demonstrated that a 12-week stationary bicycle program can alleviate depressive symptoms in individuals with mild to moderate depression, with the VR-based intervention providing superior benefits. While no significant difference was observed between the moderate-intensity and high-intensity cycling in terms of depression improvements, moderate-intensity cycling was associated with greater satisfaction. These findings suggest that integrating VR into stationary cycling may enhance exercise adherence and therapeutic outcomes for depression, and future interventions should explore VR customization and long-term effects to optimize its clinical application.

## Supplementary material

10.2196/72021 Multimedia Appendix 1 Comparison of participants with complete data and participants with missing data at 12 weeks due to loss to follow-up or withdrawal.

10.2196/72021 Checklist 1 CONSORT-EHEALTH (Consolidated Standards of Reporting Trials of Electronic and Mobile Health Applications and Online Telehealth) checklist.

## Abbreviations

CONSORT-EHEALTH: Consolidated Standards of Reporting Trials of Electronic and Mobile Health Applications and Online Telehealth
ES: effect size
HAMD-17: Hamilton Depression Rating Scale
HR_max_: maximum heart rate
*ICD-10*: *International Statistical Classification of Diseases and Related Health Problems, 10th Revision*
ITT: intention-to-treat
VR: virtual reality
