# Supplementary material for: Stationary Cycling Exercise With Virtual Reality to Reduce Depressive Symptoms Among People With Mild to Moderate Depression: Randomized Controlled Trial
Source: J Med Internet Res. 2025 Jul 15;27:e72021. doi: 10.2196/72021 (PMC12283064; doi:10.2196/72021)
Supplement: Multimedia Appendix 1 [file jmir-v27-e72021-s001.docx]

**Table S1.** Comparison of participants with complete data and participants with missing data at 12 weeks due to loss to follow-up or withdrawal.

|  | Participants with completed the study  (N=101) | Participants who were lost to follow-up or withdrawal  (N=13) | χ^2^/Z | *P value* |
| --- | --- | --- | --- | --- |
| **Age, years** | 34(13.00) | 34(16.50) | 0.24 | 0.81 |
| **Sex** |  |  | 1.18 | 0.28 |
| Male | 46(45.5) | 8(62) |  |  |
| Female | 55(54.5) | 5(39) |  |  |
| **Occupation** |  |  | 0.77 | 0.70 |
| Workers and peasants | 44(43.6) | 6(46) |  |  |
| Enterprises and governments | 23(22.8) | 4(31) |  |  |
| Other | 34(33.6) | 3(23) |  |  |
| **Education** |  |  | 1.42 | 0.70 |
| Primary school and below | 24(23.8) | 5(39) |  |  |
| Junior school | 26(25.7) | 3(23) |  |  |
| Senior school | 34(33.7) | 3(23) |  |  |
| College and above | 17(16.8) | 2(15) |  |  |
| **Marital status** |  |  | 0.32 | 0.85 |
| Married | 55(54.5) | 6(46) |  |  |
| Unmarried | 39(38.6) | 6(46) |  |  |
| Divorced or widowed | 7(6.9) | 1(8) |  |  |
| **Length of diagnosed depression** | 3(2) | 3(4) | 0.56 | 0.58 |
| **Escitalopram oxalate (mg/d)** | 15(5.00) | 15(7.50) | 0.06 | 0.95 |
| **HAMD_17_** | 21(5.00) | 23(5.50) | 0.48 | 0.63 |
